# The Association of an Early Net Ultrafiltration Rate and 28-Day Mortality in Patients Receiving Continuous Kidney Replacement Therapy

**DOI:** 10.3389/fmed.2021.766557

**Published:** 2021-12-02

**Authors:** Buyun Wu, Yining Shen, Yudie Peng, Changying Xing, Huijuan Mao

**Affiliations:** Department of Nephrology, Jiangsu Province Hospital/The First Affiliated Hospital of Nanjing Medical University, Nanjing, China

**Keywords:** continuous kidney replacement therapy, net ultrafiltration, mortality, critically ill, intensive care unit

## Abstract

**Background:** An early net ultrafiltration (NUF) rate may be associated with prognosis in patients receiving continuous kidney replacement therapy (CKRT). In this study, we tested whether high or low early NUF rates in patients treated with CKRT were associated with increased mortality.

**Methods:** We conducted a retrospective, observational study among all patients in the Medical Information Mart for Intensive Care IV database who received CKRT for more than 24 h within 14 days after intensive care unit admission. We defined the early (initial 48 h) NUF rate as the amount of fluid removal per hour adjusted by the patients' weight and took it as a classified variable (low rate: <1.6, moderate rate: 1.6–3.1 and high rate: > 3.1 ml/kg/h). The association between 28-day mortality and the NUF rate was analyzed by logistic regression and mediation analyses.

**Results:** A total of 911 patients were included in our study. The median NUF rate was 2.71 (interquartile range 1.90–3.86) ml/kg/h and the 28-day mortality was 40.1%. Compared with the moderate NUF rate, the low NUF rate (adjusted odds ratio 1.56, 95% CI 1.04–2.35, *p* = 0.032) and high NUF rate (adjusted odds ratio 1.43, 95% CI 1.02–2.01, *p* = 0.040) were associated with higher 28-day mortality. The putative effect of high or low NUF rates on 28 day mortality was not direct [adjusted average direct effects (ADE) for a low NUF rate = 0.92, *p* = 0.064; adjusted ADE for a high NUF rate = 1.03, *p* = 0.096], but mediated by effects of the NUF rate on fluid balance during the same period [adjusted average causal mediation effects (ACME) 0.96, *p* = 0.010 for a low NUF rate; adjusted ACME 0.99, *p* = 0.042 for a high NUF rate]. Moreover, we found an increase trend in the NUF rate corresponding to the lowest mortality when fluid input increased.

**Conclusion:** Compared with NUF rates between 1.6–3.1 ml/kg/h in the first 48 h of CKRT, NUF rates > 3.1 and <1.6 ml/kg/h were associated with higher mortality.

## Introduction

Fluid overload (FO), defined as an absolute increase in total body volume or a relative increase in the percentage of extracellular volume over the isovolumic status of the patient, is a common complication of all emergencies. It occurs in more than 1/3 of critically ill patients and about 2/3 of patients with acute kidney injury (AKI) who need kidney replacement therapy (1, 2), and is associated with adverse outcomes (3, 4). For patients with oliguric AKI in whom diuretic treatment is ineffective, international practice guidelines recommend using of ultrafiltration for fluid management (5, 6). Ultrafiltration, defined as fluid removal during kidney replacement therapy, has been used to treat patients with AKI and FO. Observational studies have found that ultrafiltration was able to prevent the deterioration of FO, thereby reducing the risk of death (7).

The net ultrafiltration (NUF) rate is used to denote the rate at which extracellular fluid volume is removed from the patient per unit of time during ultrafiltration (7). The NUF rate range commonly used in clinical practice is 0–10 ml/kg/h. A high NUF rate represents an increased capacity to get fluid balance and eliminate FO but may exceed vascular refilling capacity and lead to hypotension. In contrast, a low NUF rate may not associate with significant hemodynamic stress but might prolong exposure to tissue edema and organ dysfunction in patients with FO. To date, the ideal NUF rate has not been determined in clinical practice due to the lack of high-quality data (8). Previous studies indicated that a low NUF rate (<1.01 ml/kg/h) was associated with decreased survival (7, 9, 10). A high NUF rate (>1.75 ml/kg/h) was also related to increased mortality (7, 11–13). Based on these studies, Murugan et al. recently proposed that the relationship between the NUF rate and mortality in critically ill patients receiving continuous kidney replacement therapy (CKRT) might be “J” type and that a NUF rate of 1.01–1.75 ml/kg/h might be the optimal range (7). However, the data of two previous studies were obtained over 10 years ago (9, 11); thus, it may not reflect current usual practice. Besides, accounting for the unstable hemodynamics, the NUF rate was usually set to acquire fluid balance and depletion of FO, which is mainly influenced by fluid input (5); patients with higher fluid input may need a higher NUF rate. Considering the extensive use of CKRT globally, the proposed optimal NUF rate is urgent to be confirmed or revised further.

Here, we analyzed the association between the early NUF rate and 28-day mortality in patients receiving CKRT, and we hypothesized that a different optimal range of the NUF rate might exist.

## Materials and Methods

### Source of Data

Our study was based on a public critical care database in the United States named the Medical Information Mart for Intensive Care IV (MIMIC-IV) version 0.4 (14). The MIMIC-IV recorded the demographic data, vital signs, medications, laboratory tests, and other essential data of 383,220 adult admissions to the intensive care unit (ICU) in the Beth Israel Deaconess Medical Center (Boston, MA, USA) from 2008 to 2019. The establishment of the MIMIC-IV database was approved by the Massachusetts Institute of Technology (Cambridge, MA) and the Institutional Review Boards.

Our study was conducted entirely on publicly available, anonymized data; thus, individual patient consents were waived. Because this study was an analysis of third-party anonymous public databases, we completed the National Institutes of Health's web-based course and passed the Protecting Human Research Participants exam (no.38540012 and no.32559175) to apply for access to the database.

### Study Population

A total of 2,360 patients who received CKRT were recorded in the MIMIC-IV database. Patients included in this analysis were those who: (1) started CKRT treatment within 14 days of ICU admission; and (2) had a CKRT duration time of over 24 h. Patients who met the following criteria were excluded: (1) patients who died within 48 h of ICU admission; (2) end-stage kidney disease patients; (3) patients who received intermittent hemodialysis or plasmapheresis; and (4) patients with incomplete important confounders data or ultrafiltration data in the first 48 h of CKRT. Incomplete ultrafiltration data refers to any of the following: the lack of weight adjusted by the hourly NUF rate in the first 48 h of CKRT, FO percentage (defined as a positive value of total fluid input minus total fluid output, adjusted based on the patients' weight in kilograms) before CKRT, overall FO percentage 48 h after the start of CKRT and mean arterial pressure or Vasoactive-inotropic Score (VIS); and (5) patients with an NUF rate > 7.49 ml/kg/h.

### Data Extraction

Data for each patient were extracted from the MIMIC-IV database by the Structured Query Language in PostgreSQL (version12) (15). We extracted demographic characteristics (age, sex, race, height, weight, type of admission, and type of ICU), complications (hypertension, diabetes mellitus, chronic kidney disease, chronic heart failure, cancer, and Charlson Comorbidity Index Score), the severity of the illness [Oxford Acute Severity of Illness Score (OASIS)] (16) on the first day of admission, sequential organ failure assessment (SOFA) score (17) before CKRT, VIS (18) before CKRT, laboratory tests, vital signs, CKRT data (including CKRT settings, ultrafiltration data, fluid balance and intervals from ICU admission to CKRT) and clinical outcomes, among others.

For repeated measurement data, we evaluated the maximum and minimum values at the same time. We did not attempt to estimate the sample size of the study and included all eligible patients in the database to maximize the statistical power of the predictive model. As missing data may deviate from the analysis, we used multiple interpolations to deal with the missing data of body mass index (BMI), which demonstrated a missing ratio of 18.9% (172/911).

### Definitions

Sepsis was defined as life-threatening organ dysfunction caused by a dysregulated host response to infection, in which organ dysfunction can be identified as an acute change in the total SOFA score ≥ 2 points consequent to the infection (19). VIS was used to objectively quantify the degree of hemodynamic support and reflects vasoactive drugs' dosage (18). The definition of baseline serum creatinine was defined by the following rules: (i) if the lowest creatinine value during this admission was normal (<1.1 mg/dl), then we used the value; (ii) if the patient was diagnosed with chronic kidney disease, we used the lowest creatinine value during ICU stay, although in some cases it was rather high; and (iii) otherwise, we estimated the baseline serum creatinine using serum creatinine calculated from the simplified modification of diet in renal disease equation set to 75 ml/min per 1.73 m^2^.

### Exposures

The primary exposure was the NUF rate during the first 48 h of CKRT, defined as the volume of NUF removed per hour, adjusted by the patients' weight in kilograms. We calculated the NUF rate using the following equation: NUF rate (in milliliters per kilogram per hour) = cumulative NUF volume at the end of 48 h (in milliliters)/[weight at the beginning of CKRT (in kilograms) × treatment duration in the first 48 h (in hours)] (11).

### Primary Endpoint

The primary outcome was 28-day mortality. Patients discharged from the hospital alive before 28 days were considered alive at Day 28.

### Statistical Analyses

Categorical variables were presented as numbers and percentages and compared by χ^2^ tests. Continuous variables were presented as medians and interquartile ranges and compared using the Wilcoxon rank-sum test.

First, we confirmed that the relationship between the NUF rate and 28-day mortality was nearly U-shape according to the multivariate generalized additive linear model. By adding 5% (taking as a clinically acceptable and meaningful boundary value) on minimum 28-day mortality, we arrived at a new mortality rate as a cutoff value, which response to two cutoff values of NUF rate. We then classified patients into three groups using these two cutoff values.

We used Kaplan–Meier survival plots with the log-rank test to compare mortality over the first 28 days among the groups. We applied univariate and multivariate logistic regression models with the NUF rate in the first 48 h as a categorical variable to evaluate the relationship between the NUF rate and 28-day mortality. The confounding factors taken into account in this model as continuous variables included age (year), BMI (kg/m^2^), baseline serum creatinine (mg/dl), Charlson Comorbidity Index Score (point), OASIS on the first day of admission (point), time from ICU admission to the initiation of CKRT (days), mean arterial pressure before CKRT (mmHg), VIS before CKRT (point), SOFA score before CKRT (point), FO percent before CKRT (%) and cumulative FO percent in the first 48 h of CKRT (%). The confounding factors as grouping variables included male gender or not, ICU type (cardiovascular ICU or other), sepsis or not on the first day of admission, need of mechanical ventilation or not on the first day of admission. The above confounding factors was primarily consistent with the previous publication (9–12), in which CKRT durations was excluded because it should be taken as an outcome variable.

We assessed the robustness of the findings through multiple sensitivity analyses. First, a logistic regression model was used to evaluate the relationship between the NUF rate in the first 48 h of CKRT and hospital mortality. Second, as the potential impact of the NUF rate categories on the primary outcome of 28-day mortality violated the proportional hazards assumption (Supplementary Figure 1), we applied a Gray piecewise-constant time-varying coefficients model (20, 21), which considered the confounders as mentioned above. We estimated risk-adjusted hazard ratios with 95% confidence interval (CI) at five time-intervals and four nodes. The number of time intervals was selected based on prior work (9, 11, 12), and the duration of each time interval was selected by the model to ensure approximately equal distribution of deaths within each time interval (22).

In order to explore why our ultrafiltration range was different from that of others, we divided the fluid input/percentage of FO within the first 48 h of CKRT into three groups according to the tertiles and divided the NUF rate into nine groups according to the eighth percentile. Then, we calculated the 28-day mortality of the three groups with different fluid input/percentage of FO in different NUF rate ranges.

We applied a multivariable mediation model (23–25) to investigate whether the association of the NUF rate with mortality was modulated by its effect with cumulative FO percent in the first 48 h of CKRT as a mediator. The following estimates were described: (1) the total effect (estimate of the total putative effect of the NUF rate on 28-day mortality); (2) the average causal mediation effect (ACME), a variable that explains how much of the putative effect of the NUF rate on mortality was explained by the possible effect of the mediator; and (3) the average direct effect (ADE), a variable that explains how much of the putative effect of the NUF rate on mortality is still explained by the rate after considering the effect of any given mediator.

All hypothesis tests were two-tailed with a *p*-value of <0.05 considered statistically significant. All analyses were performed using R software, version 4.0.3 (26).

## Results

### Study Population

**Figure 1:**
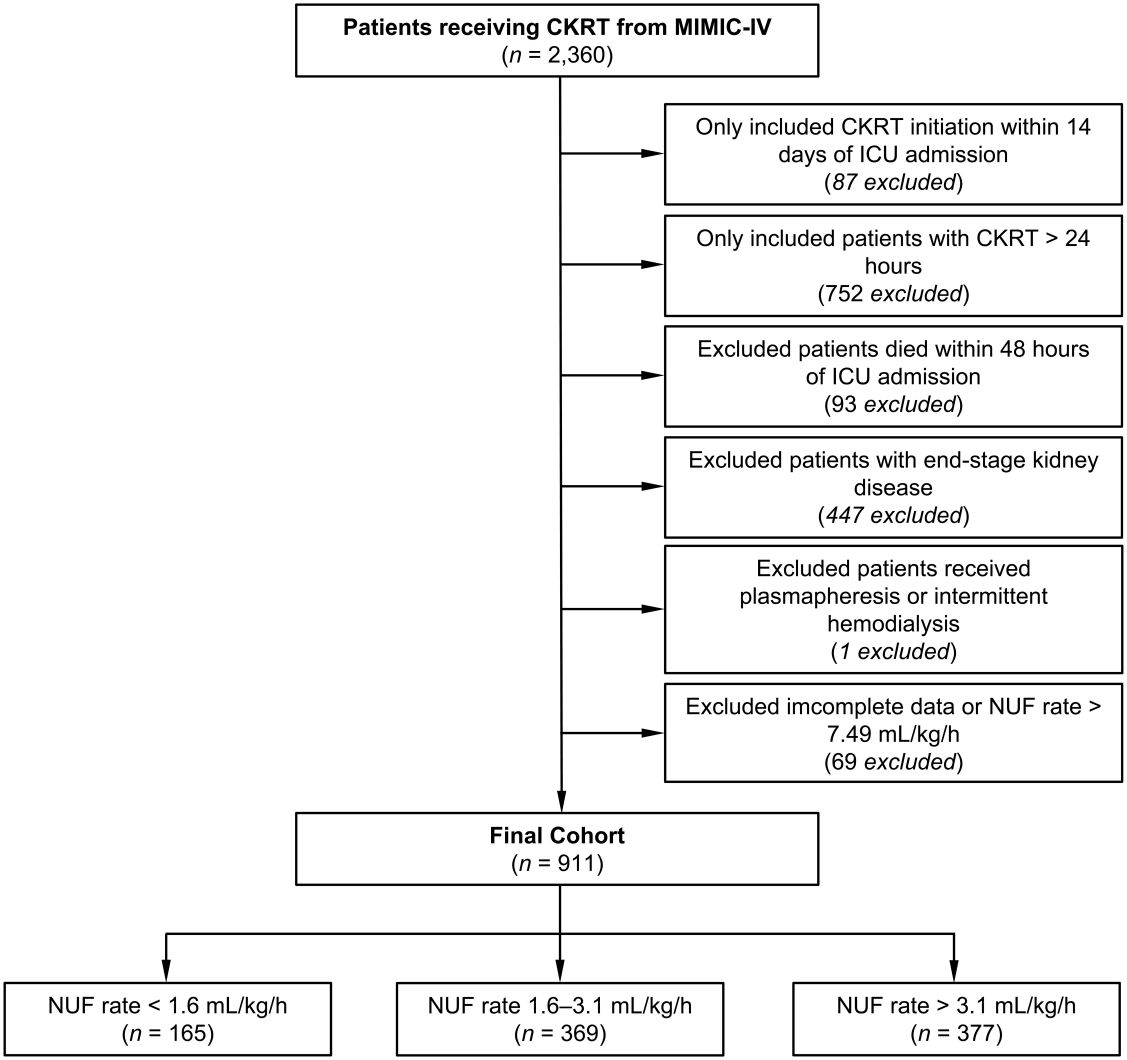
Flowchart of the study. CKRT, continuous kidney replacement therapy; ICU, intensive care unit; MIMIC-IV, Medical Information Mart for Intensive Care IV; NUF, net ultrafiltration.

**Figure 2:**
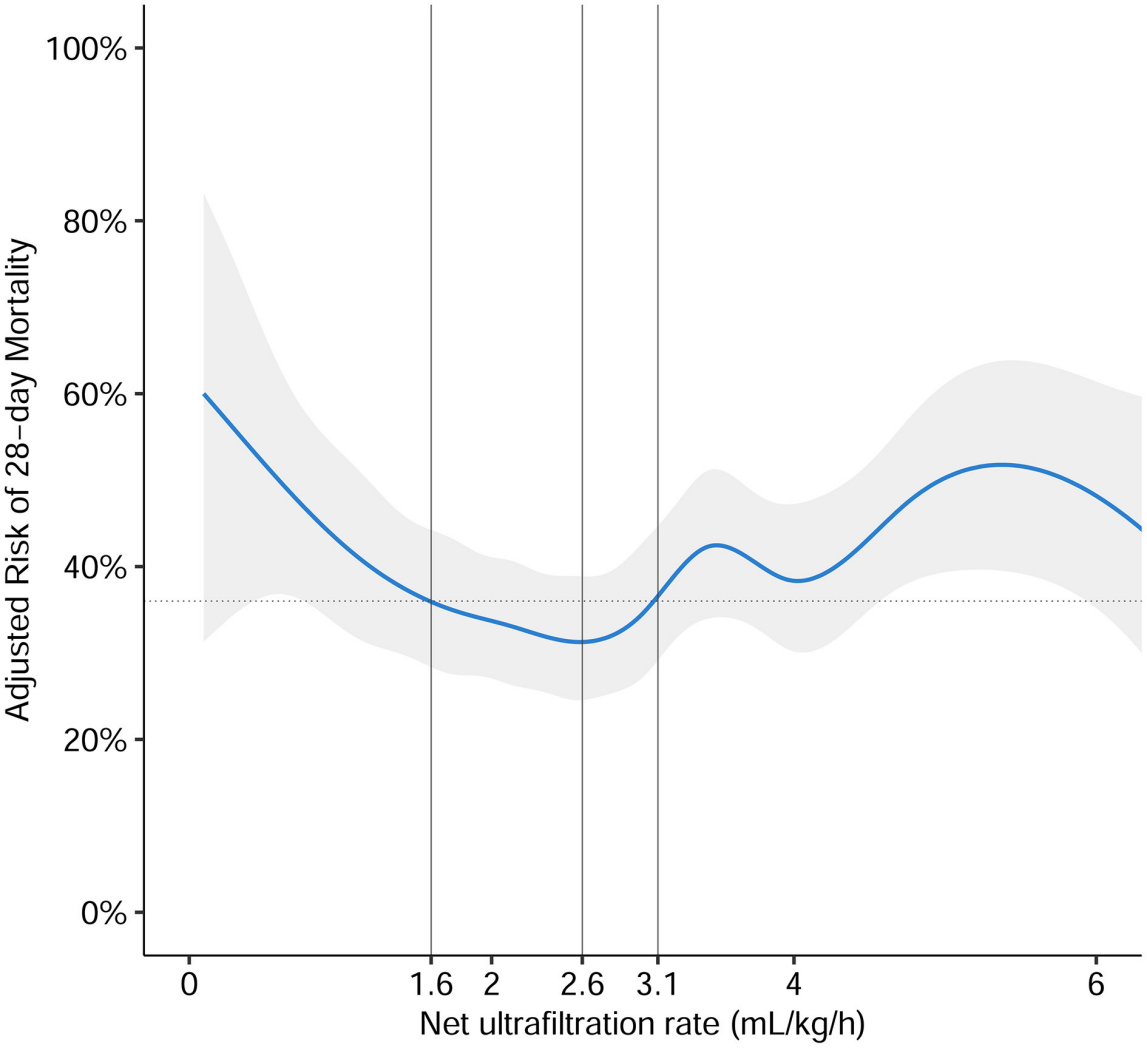
The association of NUF rate and risk of 28-day mortality. The association was plotted using a multivariate generalized additive linear model, which accounts for age, gender, body mass index, ICU type, baseline serum creatinine, Charlson Comorbidity Index Score, Oxford Acute Severity of Illness Score on the first day of admission, sepsis on the first day of admission, need of mechanical ventilation on the first day of admission, time from ICU admission until start of CKRT in minutes, mean arterial pressure before CKRT, Vasoactive-Inotropic Score before CKRT, sequential organ failure assessment score before CKRT, fluid overload percent before CKRT, and cumulative fluid overload percent in the first 48 h of CKRT. According to the multivariate generalized additive linear model, we found that the minimum 28-day mortality was 31%, which corresponded to the NUF rate of 2.6 ml/kg/h (gray solid line). By adding 5% (taking as an acceptable boundary value), we arrived at a mortality rate of 36% as a cutoff value (gray dotted lines), which corresponded to NUF rates of 1.6 and 3.1 ml/kg/h (gray solid lines). Then we stratified the NUF rate into three groups: low (<1.6 ml/kg/h), moderate (1.6–3.1 ml/kg/h), and high (>3.1 ml/kg/h). The blue solid line represented the relationship between the NUF rate in the first 48 h of CKRT and 28-day mortality and the gray shadow represents the 95% confidence interval.

Following the inclusion and exclusion criteria, we included 911 patients from the MIMIC-IV database (Figure 1). According to the multivariate generalized additive linear model, we found that the minimum 28-day mortality was 31%. By adding 5% (taking as a clinically acceptable boundary value) on this basis, we arrived at a mortality rate of 36% as a cutoff value, which corresponded to an NUF rate of 1.6 and 3.1 ml/kg/h. Then, we stratified the NUF rate into three groups: low (<1.6 ml/kg/h), moderate (1.6-3.1 ml/kg/h) and high (>3.1 ml/kg/h) (Figure 2). Among 911 patients, 165 (18.1%) had an NUF rate of <1.6 ml/kg/h, 369 (40.5%) had an NUF rate of 1.6–3.1 ml/kg/h and 377 (41.4%) had an NUF rate of >3.1 ml/kg/h (Table 1).

**Table 1:** Baseline characteristics of each NUF rate group at ICU admission before CKRT.

	**NUF rate <1.6 ml/kg/h**	**NUF rate 1.6–3.1 ml/kg/h**	**NUF rate > 3.1 ml/kg/h**	***P*-value**
	**(*n* = 165)**	**(*n* = 369)**	**(*n* = 377)**	
Demographics				
Age (year)	64.0 (53.0, 73.0)	62.0 (51.0, 73.0)	64.0 (52.0, 74.0)	0.265
Male gender (%)	108 (65.5)	231 (62.6)	208 (55.2)	0.034
White Ethnicity (%)	97 (58.8)	237 (64.2)	252 (66.8)	0.321
Weight (kg)	100 (86, 120)	99 (85, 116)	83 (70, 97)	<0.001
BMI (kg/m^2^)	34.7 (29.3, 40.0)	33.8 (29.3, 40.8)	29.9 (26.1, 35.1)	<0.001
Surgical admission (%)	72 (43.6)	151 (40.9)	149 (39.5)	0.668
Cardiovascular ICU (%)	67 (40.6)	121 (32.8)	98 (26.0)	0.003
Comorbidity				
Hypertension (%)	97 (58.8)	217 (58.8)	197 (52.3)	0.146
Diabetes (%)	33 (20.0)	67 (18.2)	53 (14.1)	0.155
Chronic kidney disease (%)	63 (38.2)	138 (37.4)	171 (45.4)	0.065
Chronic heart failure (%)	80 (48.5)	143 (38.8)	139 (36.9)	0.035
Cancer (%)	17 (10.3)	34 (9.2)	38 (10.1)	0.894
Charlson Comorbidity Index Score	4 (2, 5)	3 (2, 5)	3 (2, 5)	0.154
OASIS day1	46 (40, 51)	45 (38, 51)	46 (38, 52)	0.736
Sepsis (%)	118 (71.5)	242 (65.6)	251 (66.6)	0.389
Ventilation (%)	125 (75.8)	267 (72.4)	281 (74.5)	0.661
Baseline serum creatinine (mg/dl)	1.0 (0.8, 1.1)	0.9 (0.7, 1.1)	0.9 (0.6, 1.1)	0.249
Data before CKRT				
Mean Heart rate (beats/minute)	93 (81, 108)	91 (80, 103)	89 (80, 102)	0.266
Mean arterial pressure (mmHg)	71.0 (64.0, 79.0)	72.0 (66.0, 79.0)	72.0 (66.0, 81.0)	0.151
VIS	1.00 (0.00, 2.68)	0.80 (0.00, 2.35)	0.80 (0.00, 2.18)	0.465
Vasopressor (%)	111 (67.3)	247 (66.9)	247 (65.5)	0.889
Inotropics (%)	24 (14.5)	32 (8.7)	30 (8.0)	0.044
SOFA score	13 (11, 15)	13 (11, 15)	12 (10, 14)	0.002
Urine output in 6 hours (mL)	75 (17, 256)	80 (24, 220)	69 (22, 200)	0.579
Oliguria (%)	85 (55.6)	198 (56.1)	215 (61.1)	0.321
Fluid overload (%)	46 (27.9)	115 (31.2)	167 (44.3)	<0.001
FO percent before CKRT (%)	5.2 (2.0, 10.3)	5.7 (2.1, 12.1)	8.6 (2.9, 15.0)	<0.001
Interval from admission to CKRT (day)	2.0 (0.8, 4.6)	1.9 (0.8, 3.9)	2.1 (0.9, 4.4)	0.147
Laboratory data before CKRT				
Minimum pH	7.27 (7.15, 7.35)	7.27 (7.19 7.33)	7.27 (7.19, 7.34)	0.694
Minimum PaO_2_/FiO_2_	145 (81, 206)	163 (101, 222)	158 (100, 235)	0.155
Maximum PCO_2_ (mmHg)	45 (39, 51)	44 (38, 52)	42 (36, 49)	0.013
Minimum bicarbonate(mmol/L)	17 (14, 22)	18 (14, 20)	17 (13, 21)	0.703
Maximum potassium (mmol/L)	5.0 (4.3 5.6)	5.0 (4.4, 5.7)	4.8 (4.2, 5.5)	0.018
Maximum BUN (mg/dl)	59.0 (36.0, 94.0)	64.0 (42.8, 94.0)	67.0 (44.0, 97.0)	0.304
Maximum creatinine (mg/dl)	3.8 (2.8, 5.2)	4.1 (2.8, 5.3)	3.9 (2.9, 5.0)	0.356
Minimum albumin (g/dl)	2.6 (2.2, 3.2)	2.9 (2.4, 3.4)	2.9 (2.3, 3.4)	0.179

*BMI, body mass index; BUN, blood urea nitrogen; CKRT, continuous kidney replacement therapy; FiO_2_, fraction of inspired oxygen; FO, fluid overload; ICU, intensive care unit; NUF, net ultrafiltration; OASIS, Oxford Acute Severity of Illness Score; PaO_2_, partial pressure of oxygen; PCO_2_, partial pressure of carbon dioxide; SOFA, sequential organ failure assessment; VIS, Vasoactive-inotropic Score*.

Table 1 shows the baseline characteristics of the study patients according to the NUF rate category. The group with an NUF rate <1.6 ml/kg/h was mostly male, heavier, had higher BMI, more were from the cardiovascular ICU, more were complicated by chronic heart failure, received more usage of inotropic drugs, and had higher SOFA scores. There were no differences in disease severity scores such as the Carlson score, OASIS on the first day of ICU admission, baseline creatinine level, urine volume within 6 h before CKRT, VIS before CKRT or other key baseline characteristics (such as the presence of sepsis or oliguria or the need for vasopressor or mechanical ventilation). In the group with an NUF rate <1.6 ml/kg/h, the positive value of FO before CKRT was smaller and less complicated with FO. The group with an NUF rate of 1.6–3.1 ml/kg/h was more likely to have higher maximum serum potassium.

Table 2 shows the baseline characteristics of the three groups after the beginning of CKRT and clinical outcomes. The group with a high NUF rate was more likely to require citrate anticoagulation and had more negative fluid balance. There was no significant difference among the three groups in terms of CKRT mode, duration of CKRT, lengths of hospital, and ICU stay after the start of CKRT. The group with a moderate NUF rate had lower 28-day mortality, hospital mortality and ICU mortality and was more likely to be independent of CKRT.

**Table 2:** Characteristics of the therapy and clinical outcomes.

	**NUF rate <1.6 ml/kg/h**	**NUF rate 1.6–3.1 ml/kg/h**	**NUF rate > 3.1 ml/kg/h**	***P*-value**
	**(*n* = 165)**	**(*n* = 369)**	**(*n* = 377)**	
CKRT data				
CKRT mode (%)				0.588
CVVH	15 (9.1)	30 (8.1)	22 (5.8)	
CVVHD	2 (1.2)	5 (1.4)	3 (0.8)	
CVVHDF	147 (89.1)	329 (89.2)	350 (92.8)	
SCUF	1 (0.6)	5 (1.4)	2 (0.5)	
Dose of CKRT (mL/kg/h)	25.1 (20.5, 28.4)	24.7 (20.9, 28.1)	26.6 (22.9, 30.0)	<0.001
Anticoagulation (%)				<0.001
Citrate + heparin	6 (3.6)	5 (1.4)	13 (3.4)	
Citrate	59 (35.8)	216 (58.5)	293 (77.7)	
Heparin	29 (17.6)	29 (7.9)	14 (3.7)	
None	71 (43.0)	119 (32.2)	57 (15.1)	
Duration of CKRT (hour)	137.9 (80.0, 224.0)	129.4 (70.0, 266.1)	120.0 (71.0, 212.0)	0.301
Cumulative FO percent in first 24h of CKRT (%)	0.0 (−2.0, 2.6)	−1.2 (−2.7, 0.6)	−2.1 (−3.9, 0.3)	<0.001
Cumulative FO percent in second 24h of CKRT (%)	−0.5 (−2.0, 1.4)	−1.8 (−3.2, −0.4)	−2.8 (−4.8, −1.0)	<0.001
Cumulative FO percent in first 48h of CKRT (%)	−0.6 (−3.3, 3.7)	−2.8 (−5.2, −0.2)	−4.8 (−8.1, −1.3)	<0.001
Cumulative FO percent in total CKRT (%)	−3.9 (−9.3, 3.6)	−7.7 (−13.6, −2.7)	−9.2 (−15.9, −3.1)	<0.001
NUF data in first 48h of CKRT				
Median mean NUF (mL/h)	113 (72, 146)	226 (181, 285)	350 (297, 414)	<0.001
Median prescribed NUF (mL/h)	230 (150, 350)	350 (260, 425)	410 (350, 500)	<0.001
NUF rate (mL/kg/h)	1.11 (0.75, 1.35)	2.33 (1.97, 2.67)	4.02 (3.53, 4.79)	<0.001
Outcomes				
28-day mortality after CKRT (%)	83 (50.3)	132 (35.8)	150 (39.8)	0.007
In-hospital mortality (%)	88 (53.3)	149 (40.4)	167 (44.3)	0.021
ICU mortality (%)	79 (47.9)	130 (35.2)	141 (37.4)	0.018
Length of hospital stay after CKRT (day)	13.0 (5.3, 23.8)	15.9 (9.3, 25.0)	14.7 (7.7, 26.8)	0.084
Length of ICU stay after CKRT (day)	8.9 (5.0, 15.8)	10.0 (5.4, 16.7)	9.0 (5.2, 15.9)	0.430
Length of hospital stay (day)	16.2 (8.9, 28.2)	19.7 (12.0, 29.0)	17.8 (11.7, 30.0)	0.178
Length of ICU stay (day)	12.3 (7.7, 20.3)	13.2 (8.0, 20.6)	13.0 (8.0, 20.3)	0.774
Length of hospital stay in survivors (day)	26.8 (20.0, 40.3)	23.0 (16.0, 33.3)	24.0 (16.2, 39.2)	0.164
Length of ICU stay in survivors (day)	16.2 (9.9, 26.9)	13.8 (9.0, 21.4)	13.2 (8.7, 22.8)	0.127
Independent from CKRT (%)	91 (55.2)	262 (71.0)	262 (69.5)	0.001

*CKRT, continuous kidney replacement therapy; CVVH, continuous veno-venous hemofiltration; CVVHD, continuous veno-venous hemodialysis; CVVHDF, continuous veno-venous hemodiafiltration; FO, fluid overload; ICU, intensive care unit; NUF, net ultrafiltration; SCUF, slow continuous ultrafiltration*.

### Associations Between the NUF Rate and Primary Outcome

As compared with the moderate NUF rate group, the low NUF rate group had a higher risk of 28-day mortality [unadjusted odds ratio (OR) = 1.82 (1.25–2.64); *p* = 0.002], while the high NUF rate group had a similar risk [unadjusted OR = 1.19 (0.88–1.60); *p* = 0.258] (see Table 3, Model 1). After adjusting all confounding factors, compared with the moderate NUF rate group, the low NUF rate group was significantly associated with a higher risk of 28-day mortality (adjusted OR = 1.56, 95% CI: 1.04–2.35; *p* = 0.032), and the high NUF rate group was also associated with a higher risk of 28-day mortality (adjusted OR = 1.43, 95% CI: 1.02–2.01; *p* = 0.040) (see Table 3, Model 4). More details about the models were seen in Supplementary Table 1.

**Table 3:** The association of primary outcome and NUF rate.

**Model**	**Association**	**NUF rate**		
		**1.6–3.1 ml/kg/h**	**<1.6 ml/kg/h**	**>3.1 ml/kg/h**
Model 1	OR (95% CI)	1 (reference)	1.82 (1.25-2.64)	1.19 (0.88-1.60)
	*P*-value	/	0.002	0.258
Model 2	OR (95% CI)	1 (reference)	1.79 (1.23-2.60)	1.17 (0.86-1.60)
	*P*-value	/	0.002	0.312
Model 3	OR (95% CI)	1 (reference)	1.83 (1.25-2.69)	1.21 (0.88-1.66)
	*P*-value	/	0.002	0.233
Model 4	OR (95% CI)	1 (reference)	1.56 (1.04-2.35)	1.43 (1.02-2.01)
	*P*-value	/	0.032	0.040

*CI, confidence interval; NUF, net ultrafiltration; OR, odds ratio*.

*Model 1, unadjusted model*.

*Model 2, model 1 adjusted by age, gender and body mass index*.

*Model 3, model 2 with the addition of ICU type (whether from Cardiovascular ICU), baseline serum creatinine, Charlson Comorbidity Index Score, Oxford Acute Severity of Illness Score on the first day of admission, sepsis and need of mechanical ventilation on the first day of admission*.

*Model 4, model 3 and mean arterial pressure before CKRT, sequential organ failure assessment score before CKRT, Vasoactive-inotropic Score before CKRT, fluid overload percent before CKRT, time from ICU admission until start of CKRT in minutes and cumulative fluid overload percent in the first 48 h of CKRT*.

In addition, we divided the fluid input during the first 48 h of CKRT into three groups according to the quartiles and the NUF rate into nine groups according to the eighth percentile. We then calculated the 28-day mortality of the three groups with different input in different NUF range groups. The NUF rate corresponding to the lowest mortality was 1.78–2.12 ml/kg/h for a lower tertile group, 2.52–3.00 ml/kg/h for a middle tertile group, and 3.00–3.43 ml/kg/h for an upper tertile group. With the increase of fluid input during the first 48 h of CKRT, the range of ultrafiltration corresponding to the lowest 28-day mortality also increased (Supplementary Table 2). We did not observe the phenomena that the range of ultrafiltration corresponding to lowest 28-day mortality increase when the percentage of FO before CKRT increase (Supplementary Table 3).

### Mediation Analyses

**Figure 3:**
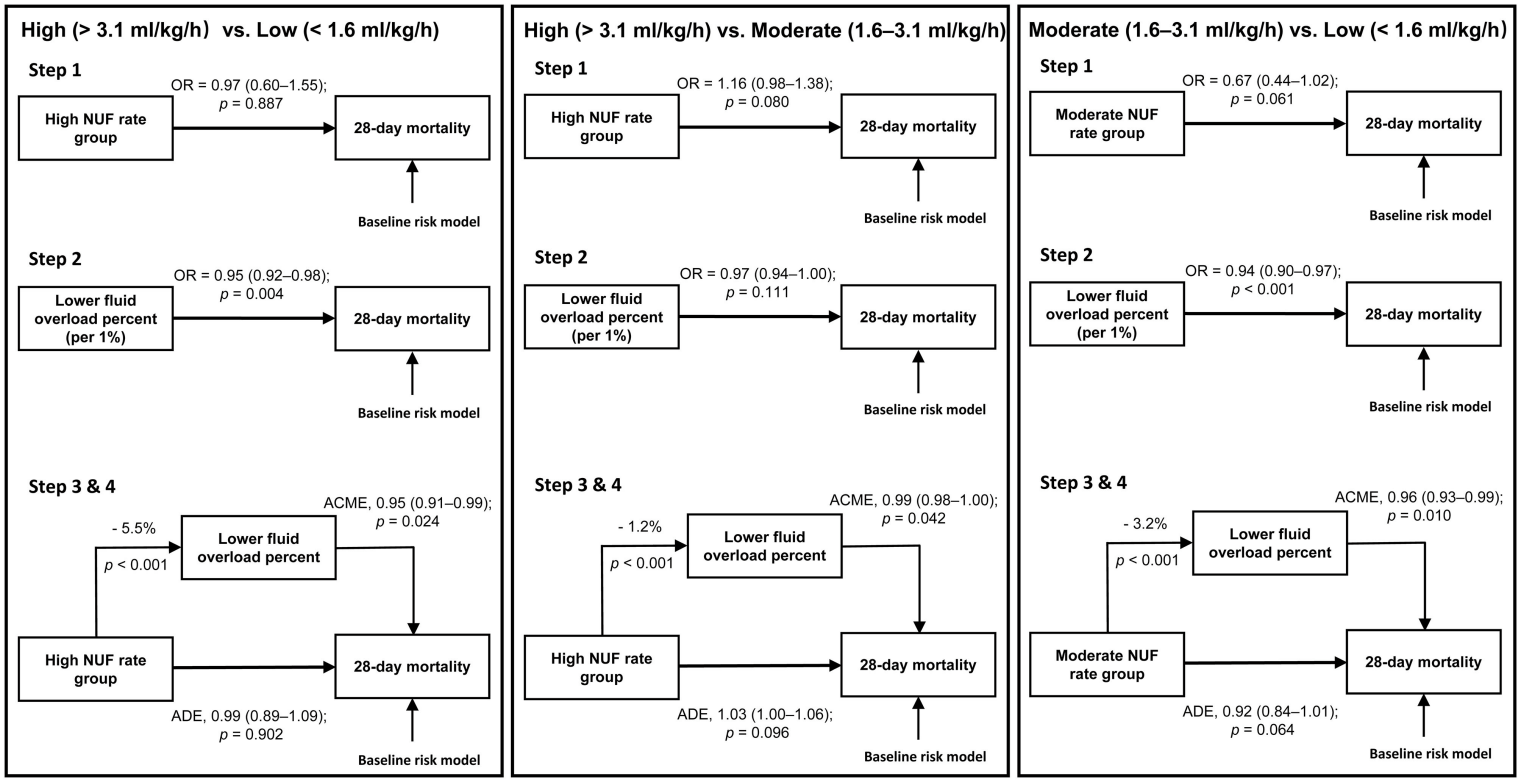
Mediation pathways for the three comparisons. ACME, average causal mediation effect; ADE, average direct effect; NUF, net ultrafiltration; OR, odds ratio. Baseline risk model included age, gender, body mass index, ICU type, baseline serum creatinine, Charlson Comorbidity Index Score, Oxford Acute Severity of Illness Score on the first day of admission, sepsis on the first day of admission, need of mechanical ventilation on the first day of admission, time from ICU admission until start of CKRT in minutes, mean arterial pressure before CKRT, Vasoactive-inotropic Score before CKRT, sequential organ failure assessment score before CKRT, fluid overload percent before CKRT and cumulative fluid overload percent in the first 48 h of CKRT. Step 1: After accounting for baseline risk factors, we applied a logistic regression model with NUF rate as a categorical variable to evaluate the relationship between the NUF rate and 28-day mortality between two of each group. Step 2: After accounting for baseline risk factors, we applied a logistic regression model with cumulative FO percent in the first 48 h of CKRT as a continuous variable to evaluate the relationship between cumulative FO percent in the first 48 h of CKRT and 28-day mortality between two of each group. Step 3: Calculation of the influence of the NUF rate group on the mediator. Step 4: After accounting for baseline risk factors, we applied a multivariable mediation model to investigate whether the association of NUF rate with mortality was modulated by its effect on cumulative FO percent in the first 48 h of CKRT as a mediator.

In adjusted mediation analyses, compared with the moderate NUF rate, the putative effect of high or low NUF rates on 28 day mortality was not direct (adjusted ADE for a low NUF rate = 0.92, 95% CI: 0.84–1.01, *p* = 0.064; adjusted ADE for a high NUF rate = 1.03, 95% CI: 1.00–1.06, *p* = 0.096) but was mediated by a causal pathway that included fluid balance during the first 48 h of CKRT (adjusted ACME for a low NUF rate = 0.96, 95% CI: 0.93–0.99; *p* = 0.010; adjusted ACME for a high NUF rate = 0.99, 95% CI: 0.98–1.00, *p* = 0.042) (Figure 3).

### Sensitivity Analyses

We used logistic regression to evaluate the relationship between the NUF rate during the first 48 h after the start of CKRT and hospital mortality. After adjusting for confounders, patients with a high NUF rate independently had a higher risk of hospital mortality than those with a moderate NUF rate (adjusted OR = 1.45, 95% CI: 1.04–2.03; *p* = 0.030) (Table 4).

**Table 4:** Analyses of the NUF rate and hospital mortality.

**NUF rate (ml/kg/h)**	**Univariable models**		**Multivariable models**	
	**Odd ratio (95% CI)**	***P*-value**	**Odd ratio (95% CI)**	***P*-value**
<1.6 ml/kg/h	1.69 (1.17-2.45)	0.006	1.46 (0.97-2.19)	0.070
1.6–3.1 ml/kg/h	1 (reference)	N/A	1 (reference)	N/A
>3.1 ml/kg/h	1.17 (0.88-1.57)	0.279	1.45 (1.04-2.03)	0.030

*Adjusted by age, gender, body mass index, ICU type, baseline serum creatinine, Charlson Comorbidity Index Score, Oxford Acute Severity of Illness Score on the first day of admission, sepsis on the first day of admission, need for mechanical ventilation on the first day of admission, time from ICU admission until start of CKRT in minutes, mean arterial pressure before CKRT, Vasoactive-inotropic Score before CKRT, sequential organ failure assessment score before CKRT, fluid overload percent before CKRT and cumulative fluid overload percent in the first 48 h of CKRT*.

The Gray model revealed that, compared with a moderate NUF rate, a low NUF rate (adjusted hazard ratio = 1.77, 95% CI: 1.20–2.61) and high NUF rate (adjusted hazard ratio = 1.25, 95% CI: 1.05–1.47) were significantly associated with a higher risk of 28-day mortality from day 5 to day 8 after the initiation of CKRT (Supplementary Table 4; Supplementary Figure 2). We demonstrated that the median early NUF rate was only beneficial during the first 5–8 days of CKRT initiation.

## Discussion

This study investigated the relationship between NUF rate and mortality in the cohort of patients receiving CKRT. After adjusting for confounding factors, we found that the NUF rates of <1.6 ml/kg/h and >3.1 ml/kg/h during the first 48 h of CKRT were associated with increased mortality compared with the NUF rate 1.6–3.1 ml/kg/h group. In addition, the optimal NUF rate range may not be in the range of 1.0–1.75 ml/kg/h (11, 12), as our results showed that 2.6 ml/kg/h correlated with the lowest risk of death, which may be due to the relatively large input in our study.

The results of this study were partly consistent with four previous studies (9–12). Murugan et al. found that in patients with volume overload >5% and receiving renal replacement therapy, the 1-year mortality in patients with an NUF rate > 25 ml/kg/d was lower than that of <20 ml/kg/d (9). Tehranian et al. found that in patients with AKI receiving CKRT, the NUF rate ≥ 35 ml/kg/d was associated with a lower 30-day mortality (10). Two other studies found that, compared with an early NUF rate of <1.01 ml/kg/h, an NUF rate of >1.75 ml/kg/h was associated with increased mortality (11, 12). Our results also supported the theory that the relationship between NUF rate and mortality in critically ill patients receiving CKRT was “J” or “U” (7), indicating that higher or lower fluid removal rates were associated with increased mortality, despite our model not being completely robust because of the limited sample size. A low NUF rate was usually set in patients with hemodynamic instability or without FO status and may be associated with prolonged exposure to tissue edema and organ dysfunction in patients with FO (27, 28). In contrast, set when patients require large fluid input or with severe FO, a high NUF rate may exceed vascular refilling capacity and associate with hemodynamic stress, leading to ischemic organ injury in critically ill patients (29, 30). Both complications could associate with decreased survival.

However, the range of the NUF rate (1.6–3.1 ml/kg/h) associated with the minimum mortality in our study was different from that in the above studies. We speculate that this is because the NUF rate depends on fluid input. If the patient had a larger fluid input, the NUF rate set by the doctor may be higher. Thus, the optimal NUF rate may be dynamic, which means it is higher when the fluid input is larger and lower when the fluid input is smaller. The optimal NUF rate still needs to be explored through further research.

Whether the putative effect of the NUF rate on mortality was direct or mediated by the fluid balance during CKRT has not been determined. Naorungroj et al. first reported that an early NUF rate >1.75 ml/kg/h was independently associated with increased hospital mortality, and the putative effect on mortality was direct, not mediated by fluid balance, but there were only 347 patients included in this study (23). Another recent large retrospective study, which included 1,434 participants, also demonstrated that in CKRT patients, compared with a moderate NUF rate (1.01–1.75 ml/kg/h), a high NUF rate (>1.75 ml/kg/h) had an ADE effect on 90-day mortality. In contrast to the results reported by Naorungroj et al. the effect of the NUF rate on mortality was also mediated by the fluid balance during CKRT (25). Our results were also partially consistent with the above second study (25). We showed that the putative effect of a high or low NUF rate on 28-day mortality was not direct but was mediated by its effect on fluid balance during the first 48 h of CKRT. The direct or indirect effect of the NUF rate on mortality needs to be determined by further studies.

We acknowledge certain limitations in this study. The study had a single-center retrospective design; therefore, it was difficult to prove the causal relationship between a high or low NUF rate and increased risk of 28-day mortality, and the result may not be applicable to other centers. Secondly, although we used multiple risk adjustments and included many potential confounders, there may be some residual confounders that were responsible for the observed association. Despite these limitations, this survey provides insight into the NUF rate prescription and practice, which may help plan future research and quality implementation initiatives. Randomized controlled trials are required to confirm whether the high or low NUF rate increases mortality in the future.

## Conclusion

In this study, as compared with an NUF rate 1.6–3.1 ml/kg/h during the first 48 h of CKRT, NUF rates of >3.1 and <1.6 ml/kg/h are associated with higher mortality. The putative effect of the NUF rate on mortality was mediated by the fluid balance during CKRT. Finally, the optimal NUF rate may rise when the fluid input increases.

## Data Availability Statement

The datasets presented in this study can be found in online repositories. The names of the repository/repositories and accession number(s) can be found below: The datasets used and/or analyzed during the current study are available at https://mimic-iv.mit.edu/.

## Ethics Statement

The Institutional Review Board of the Beth Israel Deaconess Medical Center (2001–P−001699/14) and the Massachusetts Institute of Technology (No. 0403000206) approved the use of the MIMIC database.

## Author Contributions

HM, BW, and YS designed the study. BW and YS sorted the data, analyzed the data, and drafted the manuscript. CX and HM contributed substantially to its revision. HM takes responsibility for the paper as a whole. All authors read and approved the final manuscript.

## Funding

The present study was supported by the Priority Academic Program Development (PAPD) of Jiangsu Higher Education Institutions (CN), General Project of the National Natural Science Foundation of China (81970639), and the 2017 Jiangsu Provincial Health and Health Wellness Scientific Research Project (H2017023).

## Conflict of Interest

The authors declare that the research was conducted in the absence of any commercial or financial relationships that could be construed as a potential conflict of interest.

## Publisher's Note

All claims expressed in this article are solely those of the authors and do not necessarily represent those of their affiliated organizations, or those of the publisher, the editors and the reviewers. Any product that may be evaluated in this article, or claim that may be made by its manufacturer, is not guaranteed or endorsed by the publisher.

## Abbreviations

ACME: average causal mediation effect
ADE: average direct effect
AKI: acute kidney injury
BMI: body mass index
BUN: blood urea nitrogen
CI: confidence interval
CKRT: continuous kidney replacement therapy
CVVH: continuous veno-venous hemofiltration
CVVHD: continuous veno-venous hemodialysis
CVVHDF: continuous veno-venous hemodiafiltration
FiO2: fraction of inspired oxygen
FO: fluid overload
ICU: intensive care unit
MIMIC-IV: Medical Information Mart for Intensive Care IV
NUF: net ultrafiltration
OASIS: Oxford Acute Severity of Illness Score
OR: odds ratio
PaO_2_: partial pressure of oxygen
PCO_2_: partial pressure of carbon dioxide
SCUF: slow continuous ultrafiltration
SOFA: sequential organ failure assessment
VIS: Vasoactive-inotropic Score.

## References

[1] Balakumar V Murugan R Sileanu FE Palevsky P Clermont G Kellum JA. Both positive and negative fluid balance may be associated with reduced long-term survival in the critically ill. Crit Care Med. (2017) 45:e749–57. 10.1097/CCM.0000000000002372 28437375 PMC5511076

[2] Bellomo R Cass A Cole L Finfer S Gallagher M Lee J. An observational study fluid balance and patient outcomes in the randomized evaluation of normal vs. augmented level of replacement therapy trial. Crit Care Med. (2012) 40:1753–60. 10.1097/CCM.0b013e318246b9c6 22610181

[3] Vaara ST Korhonen AM Kaukonen KM Nisula S Inkinen O Hoppu S. Fluid overload is associated with an increased risk for 90-day mortality in critically ill patients with renal replacement therapy: data from the prospective finnaki study. Crit Care. (2012) 16:R197. 10.1186/cc11682 23075459 PMC3682299

[4] Bouchard J Soroko SB Chertow GM Himmelfarb J Ikizler TA Paganini EP. Fluid accumulation, survival and recovery of kidney function in critically ill patients with acute kidney injury. Kidney Int. (2009) 76:422–7. 10.1038/ki.2009.159 19436332

[5] Rosner MH Ostermann M Murugan R Prowle JR Ronco C Kellum JA. Indications and management of mechanical fluid removal in critical illness. Br J Anaesth. (2014) 113:764–71. 10.1093/bja/aeu297 25182016

[6] Kellum JA Lameire N Aspelin P Barsoum RS Burdmann EA Goldstein SL. Kidney disease: improving global outcomes (KDIGO) clinical practice guideline for acute kidney injury. Kidney Int Suppl. (2012) 2:1–138. 10.1038/kisup.2011.35 25018921 PMC4089702

[7] Murugan R Bellomo R Palevsky PM Kellum JA. Ultrafiltration in critically ill patients treated with kidney replacement therapy. Nat Rev Nephrol. (2021) 17:262–76. 10.1038/s41581-020-00358-3 33177700

[8] Murugan R Ostermann M Peng Z Kitamura K Fujitani S Romagnoli S. Net ultrafiltration prescription and practice among critically ill patients receiving renal replacement therapy: a multinational survey of critical care practitioners. Crit Care Med. (2020) 48:e87–97. 10.1097/CCM.0000000000004092 31939807

[9] Murugan R Balakumar V Kerti SJ Priyanka P Chang CH Clermont G. Net ultrafiltration intensity and mortality in critically ill patients with fluid overload. Crit Care. (2018) 22:223. 10.1186/s13054-018-2163-1 30244678 PMC6151928

[10] Tehranian S Shawwa K Kashani KB. Net ultrafiltration rate and its impact on mortality in patients with acute kidney injury receiving continuous renal replacement therapy. Clin Kidney J. (2021) 14:564–9. 10.1093/ckj/sfz179 33623680 PMC7886538

[11] Murugan R Kerti S J Chang CH Gallagher M Clermont G Palevsky PM. Association of net ultrafiltration rate with mortality among critically ill adults with acute kidney injury receiving continuous venovenous hemodiafiltration: a secondary analysis of the randomized evaluation of normal vs augmented level (renal) of renal replacement therapy trial. JAMA Netw Open. (2019) 2:e195418. 10.1001/jamanetworkopen.2019.5418 31173127 PMC6563576

[12] Naorungroj T Neto AS Zwakman-Hessels L Yanase F Eastwood G Murugan R. Early net ultrafiltration rate and mortality in critically ill patients receiving continuous renal replacement therapy. Nephrol Dial Transplant. (2020) 35:1–8. 10.1093/ndt/gfaa142.P1460 32259841

[13] Murugan R Kerti SJ Chang CH Gallagher M Neto AS Clermont G. Association between net ultrafiltration rate and renal recovery among critically ill adults with acute kidney injury receiving continuous renal replacement therapy: an observational cohort study. Blood Purif. (2021) 1–13. 10.1159/000517281 34289471

[14] Johnson A Bulgarelli L Pollard T Horng S Celi LA Mark R. (2021). MIMIC-IV (version 1.0). PhysioNet. 10.13026/s6n6-xd9834675620

[15] Postgresql (version 12.4): The World's Most Advanced Open Source Relational Database. Berkeley, MA: The PostgreSQL Global Development Group (2020).

[16] Johnson AE Kramer AA Clifford GD. A new severity of illness scale using a subset of acute physiology and chronic health evaluation data elements shows comparable predictive accuracy. Crit Care Med. (2013) 41:1711–8. 10.1097/CCM.0b013e31828a24fe 23660729

[17] Vincent JL Moreno R Takala J Willatts S De Mendonça A Bruining H. The sofa (sepsis-related organ failure assessment) score to describe organ dysfunction/failure. On behalf of the working group on sepsis-related problems of the european society of intensive care medicine. Intensive Care Med. (1996) 22:707–10. 10.1007/BF01709751 8844239

[18] Belletti A Lerose CC Zangrillo A Landoni G. Vasoactive-inotropic score: evolution, clinical utility, and pitfalls. J Cardiothorac Vasc Anesth. (2020) 35:3067–77. 10.1053/j.jvca.2020.09.117 33069558

[19] Singer M Deutschman CS Seymour CW Shankar-Hari M Annane D Bauer M. The third international consensus definitions for sepsis and septic shock (sepsis-3). JAMA. (2016) 315:801–10. 10.1001/jama.2016.0287 26903338 PMC4968574

[20] Valenta Z Weissfeld L. Estimation of the survival function for gray's piecewise-constant time-varying coefficients model. Stat Med. (2002) 21:717–27. 10.1002/sim.1061 11870812

[21] Kasal J Jovanovic Z Clermont G Weissfeld LA Kaplan V Watson RS. Comparison of cox and gray's survival models in severe sepsis. Crit Care Med. (2004) 32:700–7. 10.1097/01.CCM.0000114819.37569.4B 15090950

[22] Gray RJ. Spline-based tests in survival analysis. Biometrics. (1994) 50:640–52. 10.2307/2532779 7981391

[23] Naorungroj T Neto AS Zwakman-Hessels L Fumitaka Y Eastwood G Murugan R. Mediators of the impact of hourly net ultrafiltration rate on mortality in critically ill patients receiving continuous renal replacement therapy. Crit Care Med. (2020) 48:e934–42. 10.1097/CCM.0000000000004508 32885938

[24] Zhang Z Zheng C Kim C Van Poucke S Lin S Lan P. Causal mediation analysis in the context of clinical research. Ann Transl Med. (2016) 4:425. 10.21037/atm.2016.11.11 27942516 PMC5124624

[25] Naorungroj T Serpa Neto A Murugan R Kellum JA Bellomo R. Continuous renal replacement therapy: the interaction between fluid balance and net ultrafiltration. Am J Respir Crit Care Med. (2021) 203:1199–201. 10.1164/rccm.202011-4097LE 33513318

[26] R (version 4.0.3): A Language and Environment for Statistical Computing. Vienna: R Foundation for Statistical Computing (2020).

[27] Flythe JE Curhan GC Brunelli SM. Disentangling the ultrafiltration rate-mortality association: the respective roles of session length and weight gain. Clin J Am Soc Nephrol. (2013) 8:1151–61. 10.2215/CJN.09460912 23493384 PMC3700694

[28] Davies SJ Brown EA Reigel W Clutterbuck E Heimbürger O Diaz NV. What is the link between poor ultrafiltration and increased mortality in anuric patients on automated peritoneal dialysis? Analysis of data from eapos. Perit Dial Int. (2006) 26:458–65. 10.1177/089686080602600410 16881341

[29] Burton JO Jefferies HJ Selby NM McIntyre CW. Hemodialysis-induced repetitive myocardial injury results in global and segmental reduction in systolic cardiac function. Clin J Am Soc Nephrol. (2009) 4:1925–31. 10.2215/CJN.04470709 19808220 PMC2798881

[30] Silversides JA Pinto R Kuint R Wald R Hladunewich MA Lapinsky SE. Fluid balance, intradialytic hypotension, and outcomes in critically ill patients undergoing renal replacement therapy: a cohort study. Crit Care. (2014) 18:624. 10.1186/s13054-014-0624-8 25407408 PMC4255668

