# Supplementary material for: The Association of an Early Net Ultrafiltration Rate and 28-Day Mortality in Patients Receiving Continuous Kidney Replacement Therapy
Source: Front Med (Lausanne). 2021 Dec 2;8:766557. doi: 10.3389/fmed.2021.766557 (PMC8674682; doi:10.3389/fmed.2021.766557)
Supplement: Supplementary file 1 [file Data_Sheet_1.docx]

**Supplementary Material**

Buyun Wu, Yining Shen, Yudie Peng, Changying Xing, Huijuan Mao

Affiliations: Department of Nephrology, The First Affiliated Hospital of Nanjing Medical University, Jiangsu Province Hospital, Nanjing, 210029, China

**Supplementary Table 1. Primary outcome: analyses of the NUF rate and 28-day mortality**

| Variables | **Model 1: Univariable models** | | **Model 4: Multivariate model** | |
| --- | --- | --- | --- | --- |
|  | **OR (95% CI)** | ***p*-value** | **OR (95% CI)** | ***p*-value** |
| **NUF rate** |  |  |  |  |
| < 1.6 mL/kg/h | 1.82 (1.25–2.64) | 0.002 | 1.56 (1.04–2.35) | 0.032 |
| 1.6­­–3.1 mL/kg/h | 1 (reference) | N/A | 1 (reference) | N/A |
| > 3.1 mL/kg/h | 1.19 (0.88–1.60) | 0.258 | 1.43 (1.02–2.01) | 0.040 |
| **Demographic characteristics** |  |  |  |  |
| Age (year) | 1.01 (1.00–1.02) | 0.014 | 1.02 (1.00–1.02) | 0.006 |
| Male gender | 1.23 (0.94–1.62) | 0.135 | 1.29 (0.95–1.76) | 0.102 |
| BMI (kg/m^2^) | 1.00 (0.98–1.01) | 0.685 | 1.01 (0.99–1.02) | 0.350 |
| **Unit type** |  |  |  |  |
| Cardiovascular ICU | 0.91 (0.68–1.21) | 0.504 | 1.04 (0.74–1.45) | 0.822 |
| **Pre-admission renal function** |  |  |  |  |
| Baseline serum creatinine (mg/dl) | 0.88 (0.65–1.19) | 0.407 | 1.05 (0.73–1.50) | 0.796 |
| **Severity of disease** |  |  |  |  |
| Charlson Comorbidity Index Score | 1.07 (1.01–1.13) | 0.016 | 1.05 (0.99–1.12) | 0.128 |
| OASIS day1 | 1.02 (1.01–1.04) | 0.005 | 1.04 (1.02–1.06) | < 0.001 |
| Sepsis | 0.90 (0.68–1.20) | 0.490 | 0.76 (0.55–1.05) | 0.094 |
| Mechanical ventilation | 0.69 (0.51–0.94) | 0.016 | 0.38 (0.24–0.58) | < 0.001 |
| **Before CKRT initiation** |  |  |  |  |
| Mean arterial pressure (mmHg) | 0.99 (0.98–1.00) | 0.010 | 0.99 (0.98–1.00) | 0.159 |
| SOFA score (per 1 point increase) | 1.15 (1.10–1.20) | < 0.001 | 1.16 (1.10–1.22) | < 0.001 |
| VIS (per 1 point increase) | 1.06 (1.01–1.13) | 0.028 | 0.98 (0.91–1.05) | 0.628 |
| FO percent before CKRT (per 1% increase) | 1.02 (1.01–1.04) | 0.002 | 1.02 (1.00–1.04) | 0.098 |
| Interval from admission to CKRT (per 1 day increase) | 1.06 (1.02–1.11) | 0.008 | 1.07 (1.01–1.12) | 0.015 |
| **Cumulative fluid balance, mL** |  |  |  |  |
| FO percent in 48h (per 1% increase) | 1.04 (1.02–1.07) | < 0.001 | 1.04 (1.01–1.07) | 0.003 |

*Abbreviations:* *BMI*, body mass index; *CI*, confidence interval; *CKRT*, continuous renal replacement therapy; *FO*, fluid overload; *ICU*, intensive care unit; *NUF*, net ultrafiltration; *OASIS*, Oxford Acute Severity of Illness Score; *OR*, odds ratio; *SOFA*, sequential organ failure assessment; *VIS*, Vasoactive-inotropic Score.

Model 1: Univariable Model

Model 4: Adjusted by age, gender, body mass index, ICU type, baseline serum creatinine, Charlson Comorbidity Index Score, Oxford Acute Severity of Illness Score on the first day of admission, sepsis on the first day of admission, need for mechanical ventilation on the first day of admission, time from ICU admission until start of CKRT in minutes, mean arterial pressure before CKRT, Vasoactive-Inotropic Score before CKRT, sequential organ failure assessment score before CKRT, fluid overload percent before CKRT and cumulative fluid overload percent in the first 48 hours of CKRT.

**Supplementary Table 2. The lowest mortality in groups classified by tertiles of input**

| **Eighth percentile of NUF rate (mL/kg/h)** | **Tertiles of input at the first 48 hours of CKRT (mL/kg)** | | |
| --- | --- | --- | --- |
|  | 1 (28.4–129.8) | 2 (130.1–185.5) | 3 (189.7–634.3) |
| <1.20 | (20/45) 44.4% | (24/38) 63.1% | (9/18) 50.0% |
| 1.20–1.78 | (32/68) 47.0% | (8/20) 40.0% | (5/13) 38.5% |
| 1.78–2.12 | **(16/52) 30.8%** | (9/31) 29.0% | (7/15) 46.7% |
| 2.12–2.52 | (16/36) 44.4% | (13/41) 31.7% | (10/24) 41.7% |
| 2.52–3.00 | (15/46) 32.6% | **(11/41) 26.8%** | (7/22) 31.8% |
| 3.00–3.43 | (14/26) 53.8% | (17/36) 47.2% | **(9/38) 23.7%** |
| 3.43–3.97 | (7/18) 38.9% | (17/46) 37.0% | (23/37) 62.2% |
| 3.97–4.76 | (3/7) 42.8% | (10/34) 29.4% | (22/57) 38.6% |
| >4.76 | (3/6) 50.0% | (5/16) 31.2% | (33/80) 41.2% |

*Abbreviations: CKRT*, continuous renal replacement therapy; *NUF*, net ultrafiltration.

**Supplementary Table 3.** **Association of the NUF rate and survival within each time interval**

| NUF rate (mL/kg/h) | Model | 0–3 days | 3–5 days | 5–8 days | 8–14 days | 14–28 days | *p*-value |
| --- | --- | --- | --- | --- | --- | --- | --- |
| <1.6 versus1.6–3.1 | Unadjusted | 1.62 (1.03–2.54) | 1.94 (1.34–2.81) | 1.98 (1.38–2.86) | 1.39 (0.95–2.04) | 1.16 (0.74–1.84) | < 0.001 |
|  | Adjusted | 1.07 (0.66–1.74) | 1.46 (0.98–2.18) | 1.77 (1.20–2.61) | 1.38 (0.92–2.06) | 1.12 (0.69–1.82) | 0.063 |
| >3.1 versus 1.6–3.1 | Unadjusted | 0.96 (0.79–1.18) | 1.11 (0.94–1.31) | 1.20 (1.02–1.40) | 1.08 (0.93–1.27) | 1.00 (0.83–1.21) | 0.212 |
|  | Adjusted | 1.03 (0.83–1.28) | 1.18 (0.99–1.41) | 1.25 (1.05–1.47) | 1.10 (0.93–1.30) | 0.97 (0.79–1.19) | 0.095 |
| >3.1 versus <1.6 | Unadjusted | 0.59 (0.39–0.90) | 0.62 (0.42–0.92) | 0.68 (0.48–0.96) | 0.64 (0.46–0.89) | 0.69 (0.50–0.96) | 0.025 |
|  | Adjusted | 0.84 (0.49–1.44) | 0.92 (0.60–1.42) | 0.88 (0.58–1.33) | 0.97 (0.63–1.50) | 1.05 (0.62–1.79) | 0.969 |

Unadjusted hazard ratios estimated from the Gray model for association of the NUF rate with mortality for each time interval are shown. Models included five intervals and four nodes, with the default timing of nodes chosen by the statistical program based on the number of events within each interval. A hazard ratio < 1 suggests that the NUF rate is associated with lower mortality and a hazard ratio > 1 suggests that the NUF rate is associated with higher mortality within each time interval. *P*-values reported are for the ranges of hazard ratios across time intervals from the model.

Adjusted hazard ratios estimated from the Gray model for association of the NUF rate with mortality for each time interval are shown. Adjusted by age, gender, body mass index, ICU type, baseline serum creatinine, Charlson Comorbidity Index Score, Oxford Acute Severity of Illness Score on the first day of admission, sepsis on the first day of admission, need for mechanical ventilation on the first day of admission, time from ICU admission until start of CKRT in minutes, mean arterial pressure before CKRT, Vasoactive-inotropic Score before CKRT, sequential organ failure score before CKRT, fluid overload percent before CKRT and cumulative fluid overload percent in the first 48 hours of CKRT.

**Supplementary Table 4. Subgroup analyses according to the presence or absence of FO percent > 10% before CKRT**

| **Subgroup** | **NUF rate (mL/kg/h)** | **Number of Patients** | **OR (95% CI)** | | ***p*-value** |
| --- | --- | --- | --- | --- | --- |
| fluid overload | < 1.6 mL/kg/h | 328 | Adjusted | 1.18 (0.55–2.52) | 0.671 |
|  |  |  | Unadjusted | 1.28 (0.64–2.55) | 0.482 |
|  | 1.6–3.1 mL/kg/h |  | Adjusted | 1 (reference) | N/A |
|  |  |  | Unadjusted |  |  |
|  | > 3.1 mL/kg/h |  | Adjusted | 1.34 (0.78– 2.33) | 0.288 |
|  |  |  | Unadjusted | 1.22 (0.76–1.98) | 0.410 |
| without fluid overload | < 1.6 mL/kg/h | 583 | Adjusted | 1.80 (1.09–2.97) | 0.021 |
|  |  |  | Unadjusted | 2.13 (1.37–3.33) | < 0.001 |
|  | 1.6–3.1 mL/kg/h |  | Adjusted | 1 (reference) | N/A |
|  |  |  | Unadjusted |  |  |
|  | > 3.1 mL/kg/h |  | Adjusted | 1.50 (0.96–2.36) | 0.078 |
|  |  |  | Unadjusted | 1.06 (0.72–1.55) | 0.783 |

*Abbreviations:* *CI*, confidence interval; *FO*, fluid overload; *NUF*, net ultrafiltration; *OR*, odds ratio.

We divided patients into two subgroups according to the presence or absence of FO, which was defined as fluid accumulation fluid accumulation adjusted by weight before CKRT > 10%.

Adjusted by age, gender, body mass index, ICU type, baseline serum creatinine, Charlson Comorbidity Index Score, Oxford Acute Severity of Illness Score on the first day of admission, sepsis on the first day of admission, need for mechanical ventilation on the first day of admission, time from ICU admission until start of CKRT in minutes, mean arterial pressure before CKRT, Vasoactive-Inotropic Score before CKRT, sequential organ failure assessment score before CKRT, fluid overload percent before CKRT and cumulative fluid overload percent in the first 48 hours of CKRT.

**Supplementary Table 5. Subgroup analyses according to the presence or absence of FO percent > 5% before CKRT**

| **Subgroup** | **NUF rate (mL/kg/h)** | **Number of Patients** | **OR (95% CI)** | | ***p*-value** |
| --- | --- | --- | --- | --- | --- |
| fluid overload | < 1.6 mL/kg/h | 383 | Adjusted | 1.21 (0.69–2.12) | 0.509 |
|  |  |  | Unadjusted | 1.44 (0.86–2.40) | 0.160 |
|  | 1.6–3.1 mL/kg/h |  | Adjusted | 1 (reference) | N/A |
|  |  |  | Unadjusted |  |  |
|  | > 3.1 mL/kg/h |  | Adjusted | 1.26 (0.82– 1.95) | 0.294 |
|  |  |  | Unadjusted | 1.21 (0.83–1.76) | 0.329 |
| without fluid overload | < 1.6 mL/kg/h | 528 | Adjusted | 2.25 (1.20–4.24) | 0.011 |
|  |  |  | Unadjusted | 2.44 (1.41–4.24) | 0.001 |
|  | 1.6–3.1 mL/kg/h |  | Adjusted | 1 (reference) | N/A |
|  |  |  | Unadjusted |  |  |
|  | > 3.1 mL/kg/h |  | Adjusted | 1.79 (1.00–3.26) | 0.052 |
|  |  |  | Unadjusted | 1.02 (0.62–1.68) | 0.919 |

*Abbreviations:* *CI*, confidence interval; *FO*, fluid overload; *NUF*, net ultrafiltration; *OR*, odds ratio.

We divided patients into two subgroups according to the presence or absence of FO, which was defined as fluid accumulation fluid accumulation adjusted by weight before CKRT > 5%.

Adjusted by age, gender, body mass index, ICU type, baseline serum creatinine, Charlson Comorbidity Index Score, Oxford Acute Severity of Illness Score on the first day of admission, sepsis on the first day of admission, need for mechanical ventilation on the first day of admission, time from ICU admission until start of CKRT in minutes, mean arterial pressure before CKRT, Vasoactive-inotropic Score before CKRT, sequential organ failure assessment score before CKRT, fluid overload percent before CKRT and cumulative fluid overload percent in the first 48 hours of CKRT.

**Supplemental Figure 1. Survival analysis of the NUF rate and mortality in three groups**


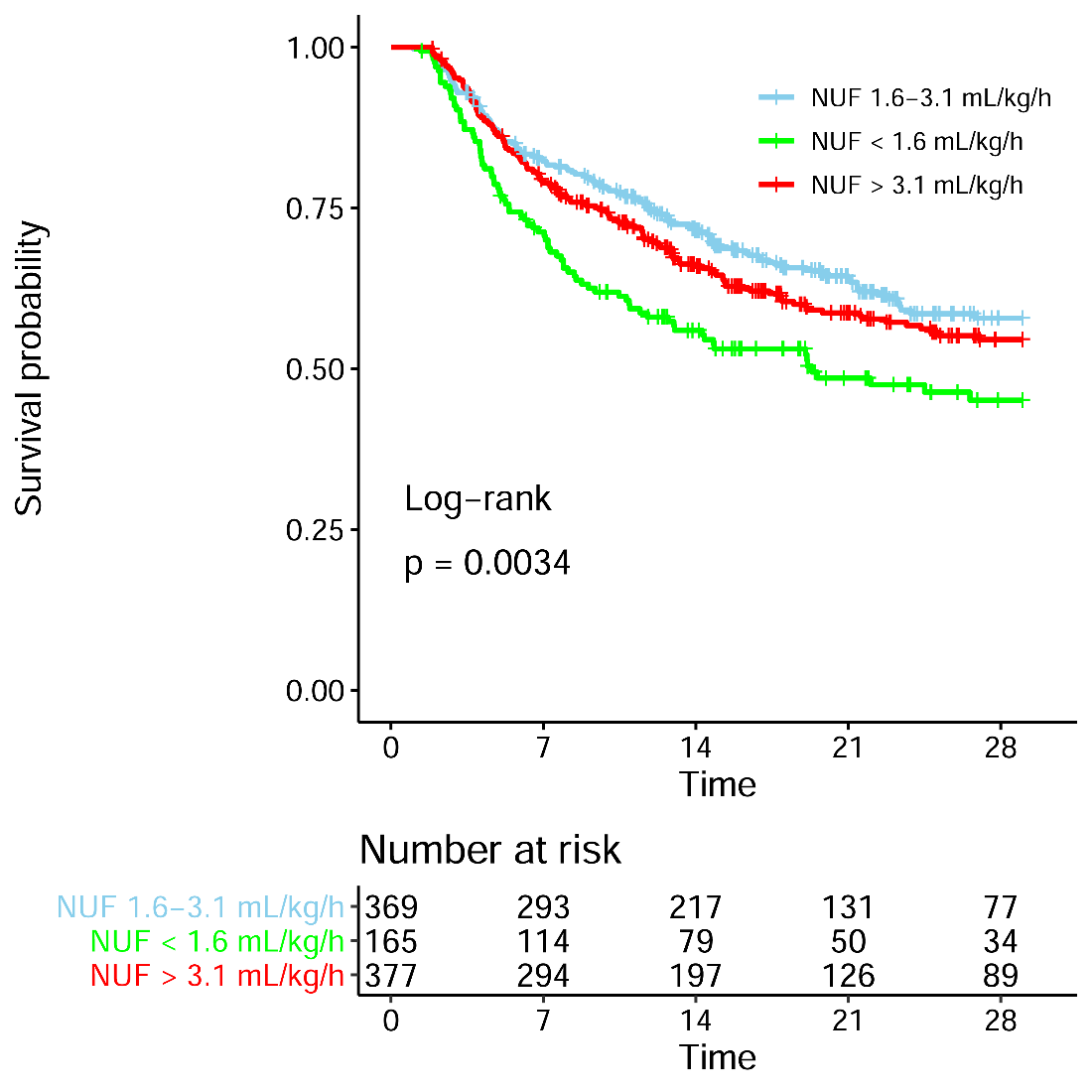


*Abbreviations:* *NUF*, net ultrafiltration.

**Supplemental Figure 2. Association of the NUF rate and survival within each time interval**


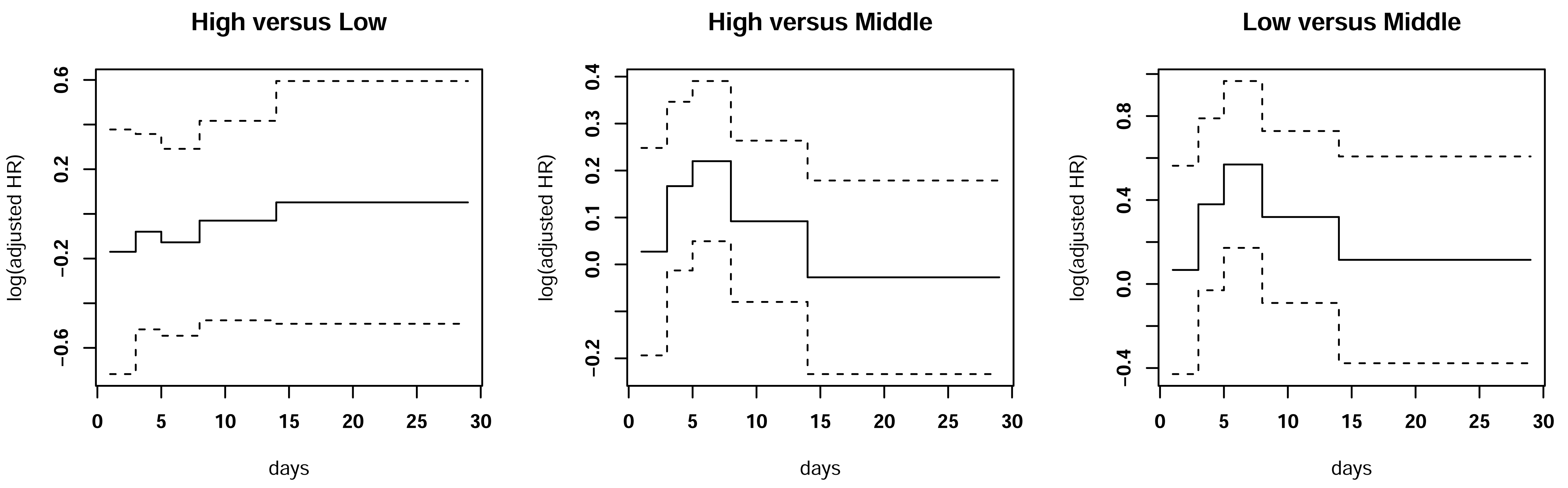
**l**

*Abbreviations:* *HR*, hazard ratio; *NUF*, net ultrafiltration.
